# Proliferation dynamics of germinative zone cells in the intact and excitotoxically lesioned postnatal rat brain

**DOI:** 10.1186/1471-2202-6-26

**Published:** 2005-04-12

**Authors:** Maryam Faiz, Laia Acarin, Bernardo Castellano, Berta Gonzalez

**Affiliations:** 1 Unit of Histology, Faculty of Medicine, Autonomous University of Barcelona, Campus UAB, 08193 Bellaterra, Spain

## Abstract

**Background:**

The forebrain subventricular zone (SVZ)-olfactory bulb pathway and hippocampal subgranular zone (SGZ) generate neurons into adulthood in the mammalian brain. Neurogenesis increases after injury to the adult brain, but few studies examine the effect of injury on neural and glial precursors in the postnatal brain. To characterize the spatio-temporal dynamics of cell proliferation in the germinative zones, this study utilized a model of postnatal damage induced by NMDA injection in the right sensorimotor cortex at postnatal day 9.

Dividing cell populations were labeled with 5-Bromodeoxyuridine (BrdU) in the intact and damaged postnatal brain. Identity of proliferating cells was determined by double immunolabeling with nestin, GFAP, NeuN and tomato lectin (TL).

**Results:**

In the control brain, grouped BrdU+ cells were observed in the Rostral Migratory Stream (RMS), SVZ and SGZ. Maximal proliferation was seen at P12, persisted until P23 and diminished by P49. After injury, a striking reduction in the number of BrdU+ cells was observed in the ipsilateral SVZ from 10 hours (58% decrease) until 14 days post-lesion (88% decrease). In contrast, an increase in grouped BrdU+ cells was seen in the striatum adjacent to the depleted SVZ. Significantly reduced numbers of BrdU+ cells were also seen in the RMS until 3 days post-lesion. No changes were noted in the SGZ. Both in controls and lesioned hemispheres, BrdU+ cells located in the germinal zones were mostly nestin positive and negative for GFAP, NeuN, and TL. In the SVZ area lining the ventricle, BrdU+/nestin+ cells were mainly located between TL+ ependyma and parenchymal GFAP+ astrocytes. After excitotoxicity, a decrease in the number and orientation of GFAP/nestin+ prolongations leaving the SVZ to the cortex, corpus callosum and striatum was noted until 5 days post-lesion.

**Conclusion:**

Postnatal excitotoxic injury differentially affects proliferating cells in the germinative zones: no change is observed in the dentate gyrus whereas excitotoxicity causes a significant decrease in proliferating cells in the SVZ and RMS. Depletion of BrdU+ cells in the postnatal SVZ and RMS differs from previous studies after adult brain injury and may affect the SVZ-RMS migration and is suggestive of progenitor recruitment to injured areas.

## Background

Progenitor cells of the central nervous system (CNS) are a population of undifferentiated cells of neuroectodermal origin with high proliferative capacity that can differentiate into neuronal cells or macroglial cells (i.e. astrocytes and oligodendrocytes).

These progenitors for neurons and glia, which can proliferate throughout life in the CNS, are located in at least 3 germinal zones: the ventricular wall and adjacent subventricular zone (SVZ), the subgranular zone of the hippocampal dentate gyrus (SGZ) and the olfactory bulb (OB) [1-3]. The fate of progenitor cells depends highly on their location; whereas progenitor cells of the SGZ migrate into the granule cell layer of the hippocampus [3], SVZ progenitors migrate tangentially in chains surrounded by astrocytic tube structures forming the rostral migratory stream (RMS) towards the OB [1]. Furthermore, progenitor cells of a more restricted fate have been described, in vitro, to reside within the cerebral cortex and provide a local source of new neurons [4]. Thus, multiple brain areas could function as reservoirs of brain precursor cells. However, the normal CNS environment is likely to limit would-be uncommitted progenitor reservoirs to a glial fate [5-7] and the role of these progenitors both in the normal and injured CNS is not well established.

After damage to the adult brain, several studies have reported different profiles of progenitor cell proliferation and differentiation, but generally show an increase of 5-Bromodeoxyuridine (BrdU)+ cells in the germinative zones of the brain [8,9]. In the SVZ, enhanced neurogenesis has been shown after aspiration lesions, inflammatory demyelization, and cortical transection lesions [10-13], although most studies have focused on the changes following focal ischemic injury, where consistent increases in the numbers of SVZ progenitor cells have been reported [13-16]. Obviously of interest is the fate and life span of newly generated progenitor cells. These cells are able to express the appropriate neuronal phenotype; however, the majority disappear by 5 weeks following the lesion [14,17]. Thus, the capacity of newly generated progenitors for CNS repair is still under debate [18]. Nevertheless, SVZ neural precursors are seen to migrate towards the primarily degenerated injury core where they are able to differentiate into neurons and glia [15]. In the SGZ of the dentate gyrus, brain insults also promote enhanced neurogenesis; increased numbers of progenitor cells have been seen after adult brain mechanical lesions, epileptic seizures, excitotoxicity and stroke [19-21]. Moreover, after global ischemia, treatment with growth factors can increase CA1 neuronal renewal and subsequent functional improvements [22].

In addition to a general injury-induced increase in progenitor cells, there are key differences in the regional proliferation and differentiation dynamics after distinct types of injury and at different ages. In the immature rat brain, it seems reasonable that postnatal dynamics of the germinative zones would be unique. A plasticity window period in the early postnatal rat brain has been defined, between days 6 and 10, and extensively studied by Kolb and his colleagues, showing that the immature cortex is capable of dendritic sprouting and cortical reorganization after injury. This plasticity results in a lack of abnormalities in cortico-cortical and subcortico-cortical connections; moreover, normal behavioral abilities are retained [23-27]. In this sense, the postnatal rat brain provides an optimal experimental model for the study of neuronal injury and the concurrent cell response to degenerative and regenerative processes. Specifically, the contribution of progenitor pools to the enhanced recovery during the plasticity window remains unknown.

To our knowledge, in spite of the fact that the post-injury environment in the developing brain differs greatly from the adult brain, there are only a few recent studies on the developmental changes of progenitor pools in the early postnatal brain and their response to brain damage, which provide contradictory data. Whereas two studies have shown decreased numbers of SVZ oligodendrocyte precursors after hypoxic-ischemic injury and a lasting decrease of neural stem cells [28,29], a more recent study has showed hypoxic-ischemic induced stimulated cell proliferation and neurogenesis in the SVZ and peri-infarct striatum [30]. Therefore, further studies based on the early postnatal stage are necessary to understand the dynamics of neurogenic and gliogenic proliferating cell populations in the developing brain and to clarify the links between developmental plasticity and neurogenesis after injury. In the most hopeful of scenarios these cells would be able to proliferate, differentiate, and integrate into injured tissue to facilitate neurological recovery from damage.

Accordingly, the aim of our study was to evaluate the temporal and spatial dynamics of cell proliferation in the three main germinative zones of the brain during normal postnatal development and following excitotoxic injury caused by an injection of N-methyl-D-aspartate (NMDA) in 9-day old rat pups. Moreover, proliferating cells in these areas were characterized by using double labeling with specific cell markers for progenitors, mature glia and neurons.

## Results

Injection of 27 ηmol of NMDA into the right sensimotor cortex of 9-day old rats caused neuronal degeneration accompanied by a glial response in the entire cortex at the level of the injection site and surrounding tissue, extending to the septum, striatum and rostral hippocampus. No affectation was seen in the contralateral hemisphere, as has been previously described in detail [31,32].

### In the intact postnatal brain, maximal proliferation is seen at P12

In the control postnatal brain, maximal proliferation was observed at postnatal day (P) 12, persisted until P16-P23 and was clearly diminished by P49. Proliferative activity was observed in the RMS, SVZ and the SGZ as well as in the cortex, striatum, thalamus, hippocampus, capsula interna, corpus callosum, and the lateral septal nuclei. In these postnatal controls, two patterns of BrdU-positive cell distribution were observed: grouped BrdU+ cells and scattered BrdU+ cells, homogeneously distributed within the brain parenchyma. Grouped BrdU+ cells, which are the focus of our study, were seen at all time points mainly in the RMS, the SVZ, and the SGZ, but were also seen until P16 in several white matter tracts (the corpus callosum, hippocampal fimbria, internal capsula, and striatal white matter). Scattered BrdU+ cells were seen in the cortex and thalamus until P16.

**Figure 1:**
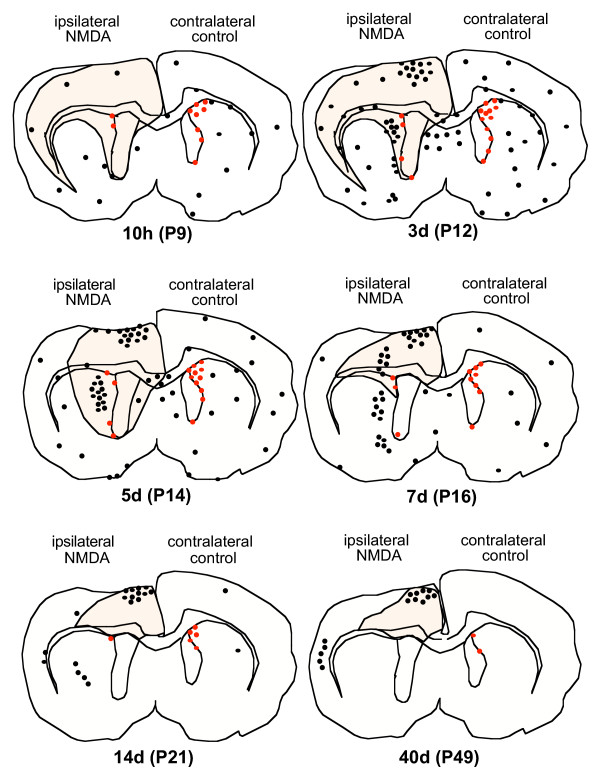
**Camera lucida drawings of anterior coronal brain sections. **Dots represent BrdU+ cells. No difference in number or spatial and temporal distribution of BrdU+ cells was seen in the contralateral and control hemispheres at corresponding time points (corresponding time points: 10 h = P9, 3d = P12, 5d = P14, 7d = P16, 14d = P23, 40d = P49). Ipsilateral (ip) hemispheres show BrdU+ cell distribution following NMDA-induced excitotoxicity in coronal brain sections at the level of the neurodegenerating area (outlined in black) and SVZ (BrdU+ cells of the SVZ are represented in red). Proliferation was observed at 10 h (P9), peaked at 3d (P12) and diminished by 40d (P49).

**Figure 2:**
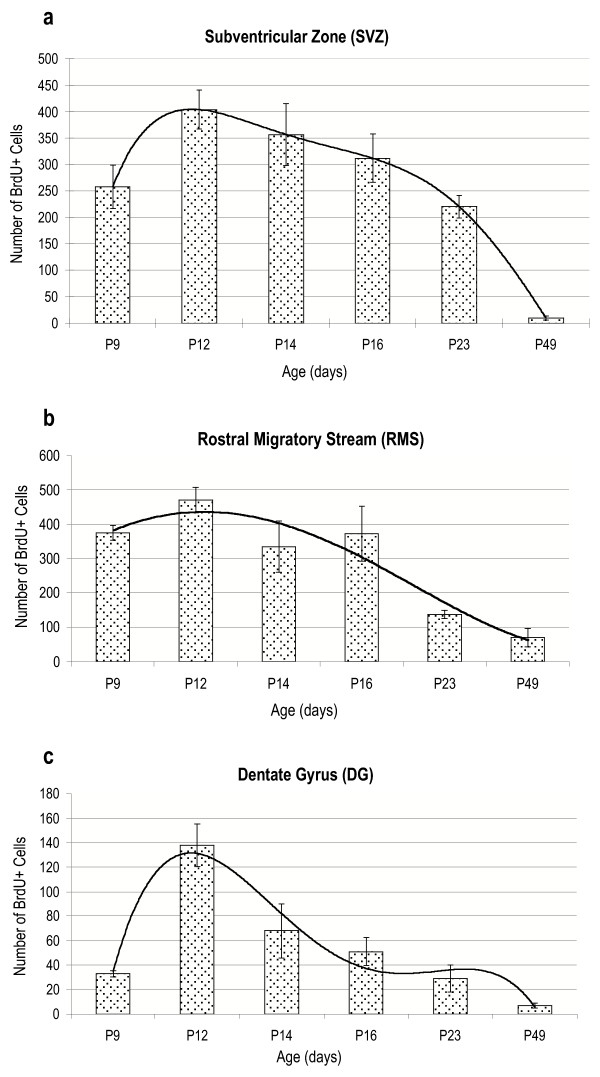
**Temporal dynamics of proliferation in the germinative zones of control brains. **Trends of cell proliferation in the postnatal rat brain, from P9 to P49, in the SVZ (a), RMS (b), and DG (c) are shown. Peak proliferation was always seen at P12. Curves were calculated using a polynomial regression line (order = 5, period = 2).

In general, in the germinative zones, BrdU+ cell numbers in control brains reached maximal numbers at P12 and then gradually decreased as the animal aged (Fig. 1). In the SVZ, a significant increase in proliferating cell number was seen from P9 to P12, numbers remained statistically constant until P23, after which a significant decrease was seen from P23 to P49 (Fig. 2a). In the RMS, a slight increase in proliferating cell numbers was seen between P9 and P12, cell numbers significantly decreased from P12 to P14, remained statistically constant until P16, and then decreased again between P16 and P23, after which no change was noted (Fig. 2b). In the DG, cell numbers significantly increased from P9 to P12, to reach maximum levels, where after numbers decreased gradually until P49 (Fig. 2c).

### Excitotoxic damage results in a reduction of BrdU+ cells in the ipsilateral SVZ and in the RMS at early survival times

In the lesioned postnatal brain, the contralateral hemisphere showed no differences in number and distribution when compared to the control postnatal brain at each corresponding time point. However, the ipsilateral damaged hemisphere showed changes in the amount and distribution of BrdU+ cells (Fig. 1). Noteworthy, were the increased amounts of BrdU+ cells observed in the neurodegenerating areas: the lesioned cortex, septum, rostral thalamus and striatum, where it was interesting to find grouped BrdU+ cells approximately at 80–100μn away from the SVZ.

**Figure 3:**
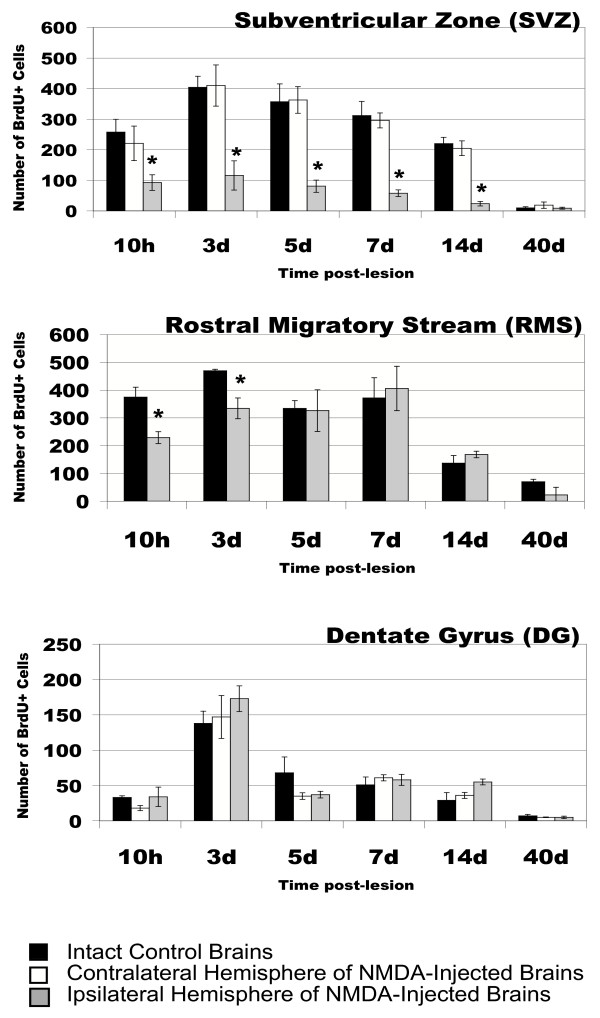
**Quantitative analysis of excitotoxicity-induced changes in the germinative zones. **Mean values of the number of BrdU+ cells in the SVZ (a), RMS (b), and DG (c) are shown and compared between intact controls, and lesioned (NMDA injected) contralateral (cl) and ipsilateral (ip) hemispheres. No difference was seen between intact controls and contralateral hemispheres at any time. In the SVZ, there is a significant decrease (asterisk) between the total number of BrdU+ cells in ip hemispheres when compared to cl and control hemispheres at all time points except 40 days post lesion. In the RMS, a significant decrease (asterisk) was seen at 10 hours and 3 days post lesion in NMDA-injected brains when compared to controls. In the DG, no significant difference in BrdU+ cell number was observed at any time point in ip hemispheres were compared to cl or control hemispheres.

**Figure 4:**
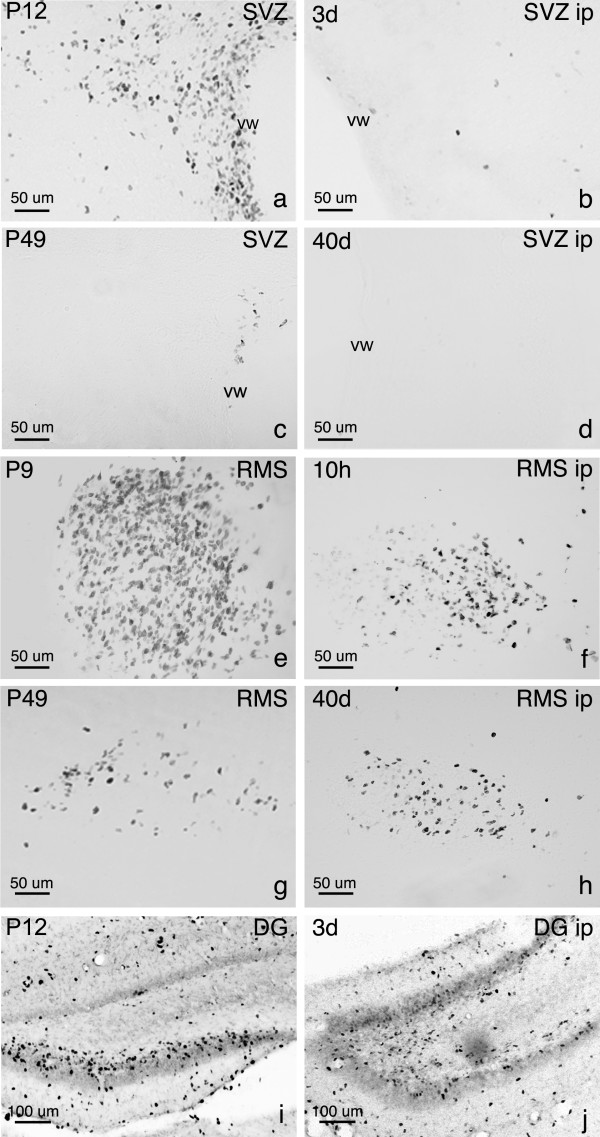
**Immunohistochemical staining for BrdU in the SVZ, RMS and SGZ. **The significant decrease in BrdU+ cell number in the ipsilateral (ip) SVZ following NMDA-induced excitotoxicity is shown in a coronal brain section at 3 days post lesion (3d; compare a, b). BrdU+ cells at all time points in the ip SVZ showed a sharp decrease in number when compared to control hemispheres until 40 days post lesion (40d), the last time point studied, where no difference was seen (c, d). In the ip RMS, a decrease in BrdU+ cells was seen at 10 hours post lesion (10 h) and at 3 days post lesion (e) when compared to the corresponding control RMS (P9 and P12 brains, respectively; f). Thereafter, the ip RMS (7–40 days post lesion) when compared to controls (P16-P49, respectively), showed no difference in BrdU+ cell numbers (g, h). The dentate gyrus of control and NMDA-injected hemispheres showed no differences in cell number at any time point, as shown in a coronal section of the DG at 3 days post lesion (i, j). Note the hippocampal shrinkage in the ipsilateral hemisphere (j).

In the ipsilateral germinal zones, changes in the number of BrdU+ cells were strongly dependent on location. In the SVZ, a striking decrease (58%) in the number of BrdU+ cells was already observed at 10 hours post lesion (Fig. 1 and Figs. 3a). At 3 days post lesion, the number of BrdU+ cells in the ipsilateral SVZ showed a 72% reduction when compared to the contralateral side, and this decrease was sustained until 14 days post lesion where an 88% decrease was seen (Fig. 3a, 4a–d). In the lesioned RMS, a significant reduction in the number of BrdU+ cells was only observed at 10 hours post lesion and 3 days post lesion whereas no changes in BrdU+ cell numbers were seen at later time points (Figs. 3b and 4e–h). In the DG of lesioned animals, although a higher density of BrdU+ cells was observed, there was notable ipsilateral hippocampal degeneration and shrinkage caused by the lesion. Because the cells were counted in the entire dentate gyrus, irrespective of area, there was no significant difference in the number of BrdU+ cells in the ipsilateral DG when compared to the contralateral control side or saline-injected controls (Figs. 3c and 4i–j).

### Most BrdU+ cells in the germinative zones express nestin. GFAP+/nestin+ prolongations leaving the SVZ in the intact postnatal brain display changes in arrangement following damage

**Figure 5:**
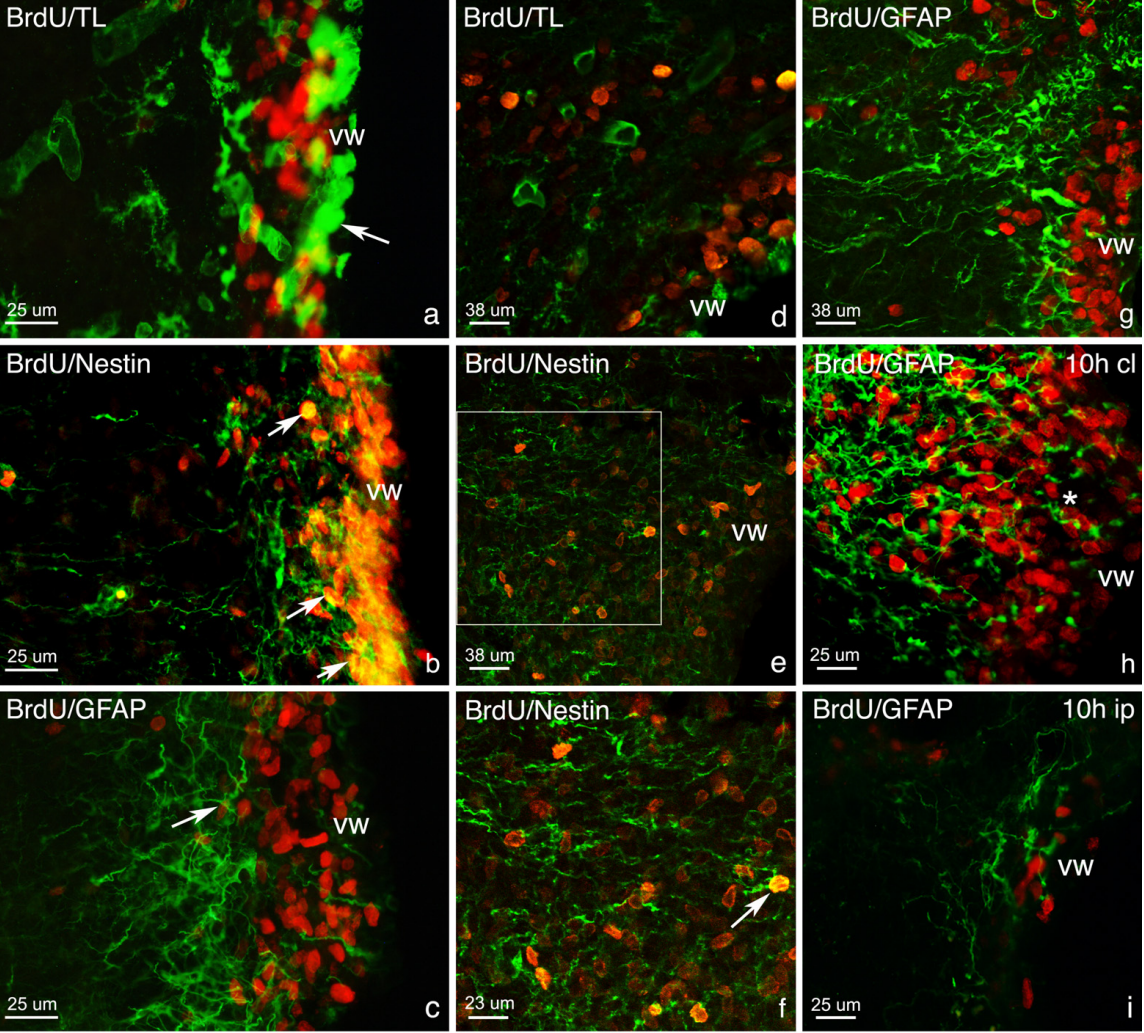
**Phenotypes of proliferating cells in the SVZ. **Optical microscope studies of coronal brain sections after double-labeled immunofluorescence showed that there is a characteristic placement of cell phenotypes along the entire length of the ventricular wall (vw). Photographs show TL+ ventricular ependymal cells (arrow, a) bordered BrdU+/nestin+ cells (arrows, b), which were found next to GFAP+ cells (arrow, c). Photographs of confocal imaging in the SVZ (more specifically, in zone a of the SVZ) revealed a similar placement where TL+ ependymal cells (d) of the ventricular wall are seen next to BrdU+/nestin+ progenitor cells (e; arrow, f) and GFAP+ cells (g). BrdU+/nestin+ cells outlined in white in photograph e can be seen at greater magnification in f. This patterning was seen at all time points except at postnatal day 9 (P9). At this time, in both control (P9) and lesioned brains (10 h), BrdU+/GFAP+ cells were also seen next to the ventricular wall (asterisk, h). At 10 hours post lesion, a decrease in the number of GFAP+ filaments in the ipsilateral (ip; i) hemisphere was also noted when compared to contralateral (cl; h) and control hemispheres. BrdU (red); nestin, TL, GFAP (green).

The study of double stained sections showed that most scattered BrdU+ cells found throughout the control brains until P16 were tomato lectin (TL)+ endothelial cells lining blood vessels (data not shown). Both in the controls and lesioned hemispheres, grouped BrdU+ cells in the germinal zones were mostly double labeled for nestin intermediate filament protein (nestin) and were negative for glial fibrillary acidic protein (GFAP) (Figs. 5a–g) and neuronal nuclear antigen (NeuN) (data not shown).

**Figure 6:**
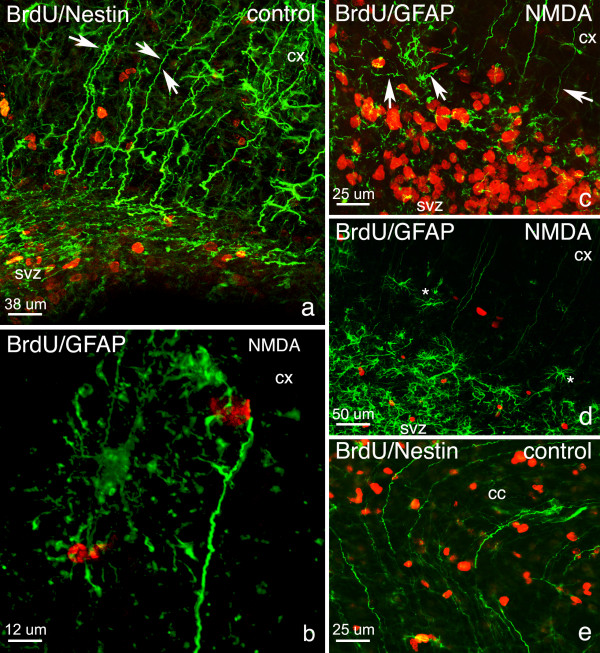
**Nestin+ and GFAP+ Projections leaving the SVZ. **In control brains at P12 and P14 GFAP+ nestin+ projections were seen leaving the SVZ extending towards the cortex (cx; arrows, a). After NMDA-induced excitotoxicity at 3 and 5 days post lesion, the presence of mature astrocytes with a star-like morphology were noticed next to the prolongations (b; asterisks, d). There was also a decrease observed in the number of prolongations (c, d) and orientation seemed disturbed such that many prolongations did not extend towards the cortex or were shorter (arrows, c) and did not reach the outer layers of the cortex (b-d). At 3 and 5 days post lesion note that the GFAP+ and nestin+ prolongations were also seen to extend into the corpus callosum (cc; e) and towards the striatum (see figure 5, b-c) in control brains at P12 and P14. BrdU (red); nestin, GFAP (green).

The SVZ area lining the ventricle showed a layered type distribution of the different cell markers. BrdU+/nestin+ cells were located between TL+ ependymal cells lining the ventricle and GFAP+ astrocytes in the parenchyma at all time points (Figs. 5a–c), except at P9, when less nestin expression was seen and GFAP+ structures were located alongside the TL+ ependymal cells of the lateral ventricle and within BrdU+ cells (Fig. 5h). In addition, until P14, GFAP+ and nestin+ prolongations were seen to extend radially from the top of the ventricle to the cortex, (Fig. 6a), curve from the SVZ to the corpus callosum (Fig. 6e), and extend from the ventricular wall to the striatum (Fig. 5b, c). These cells occasionally had a BrdU+ nucleus, and demonstrated either a bipolar morphology, or had a single prolongation leaving from the BrdU+ nucleus.

In the lesioned hemisphere, there was a decrease in the amount of prolongations leaving the SVZ when compared to the contralateral hemisphere (compare Figs. 5h and 5i). Mainly at 10 hours post lesion, but also at 3 and 5 days post lesion, there were disturbances in the orientation of GFAP+ and nestin+ prolongations (Fig. 6c, d). Moreover, at 3 and 5 days post lesion, a number of GFAP+ astrocytes were also seen alongside these prolongations (Fig. 6b, d).

**Figure 7:**
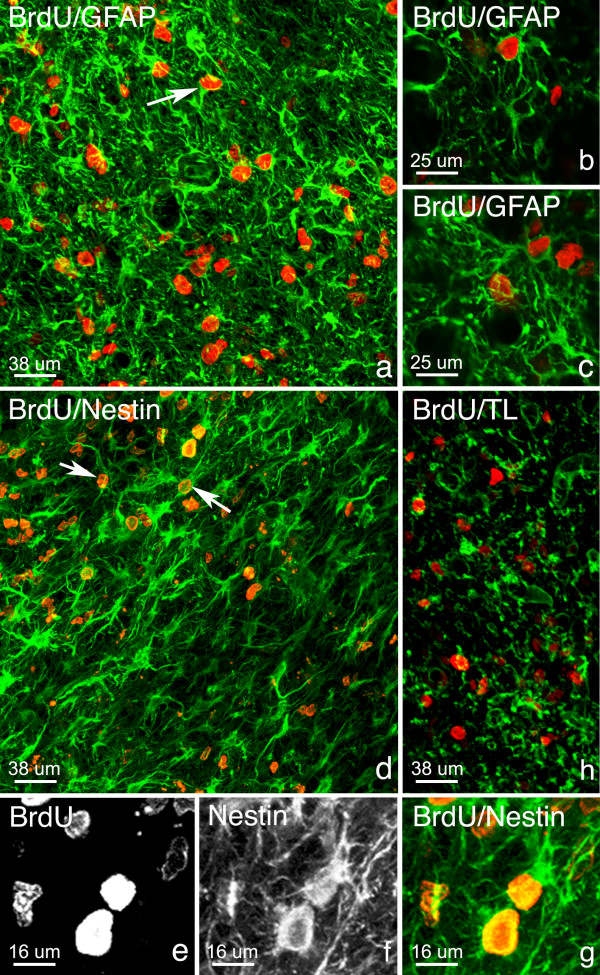
**Double immunohistochemical staining of BrdU+ cell clusters in the striatum. **In lesioned animals, from 3 days post lesion until 7 days post lesion grouped BrdU+ cells were found in the parenchyma a short distance away from the ventricular wall. Double labeling immunofluorescence studies followed by confocal imaging revealed that striatal BrdU+ cells colocalized with GFAP (arrow, a, b-c) and nestin (d, e-g). Within these groups it was possible to located cells with unipolar (arrow, a), bipolar (arrows, b), and typical mature astrocyte star-like morphology (b, c). Few BrdU+ cells were seen to colocalize with TL (h). BrdU (red); nestin, TL, GFAP (green).

As mentioned above, grouped BrdU+ cells were observed in the striatum of lesioned animals, close to the SVZ. These grouped BrdU+ cells were mainly BrdU+/GFAP+ (Fig. 7a–c) and BrdU+/nestin+ (Fig. 7d-–g). It was possible to locate mature astrocytes, with a typical star-like morphology, but also cells with a bipolar morphology and cells with few projections (Fig. 7). Additionally, a small population of BrdU+ cells in this area were TL+ (Fig. 7h).

**Figure 8:**
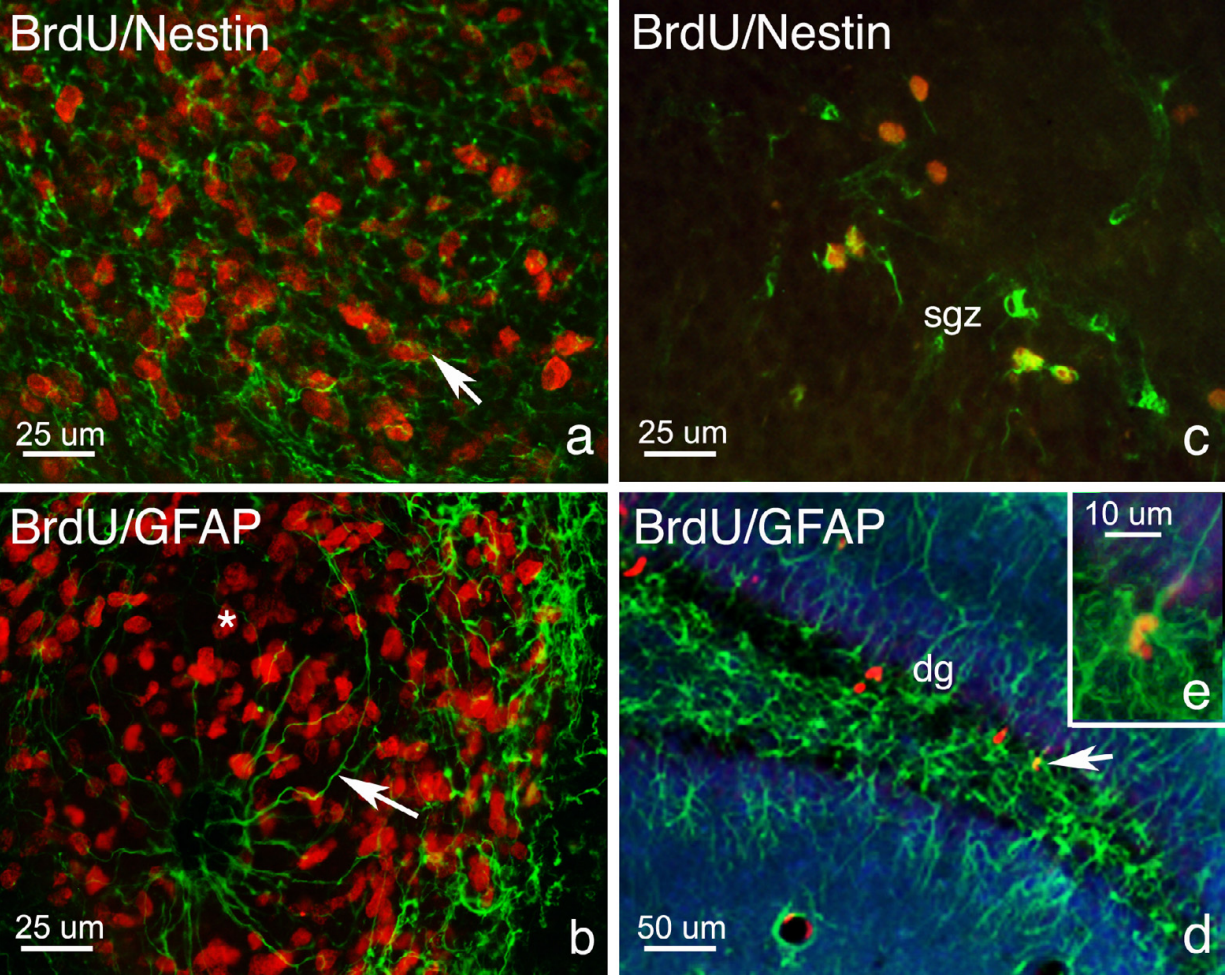
**Phenotypes of proliferating cells in the RMS and DG. **Double-labeled immunofluoresence studies showed that in the RMS most cells were BrdU+/nestin+ (arrow, a) and revealed the presence of GFAP+ filaments (arrow, b) surrounding BrdU+ cells (asterisk, b). In the DG, BrdU+/nestin+ cells (c) were seen and a few BrdU+/GFAP+ cells could also be found (arrow, d, e). BrdU (red); nestin, GFAP (green).

In the RMS, most of the BrdU+ cells were again seen to colocalize with nestin (Fig. 8a). No TL+, GFAP+, or NeuN+ cells were seen to colocalize with BrdU+ cells in the RMS. A tube-like migratory system was evident, as the BrdU+/nestin+ GFAP+ filaments surrounded cells. Especially spectacular, was the RMS at 10 hours post lesion, where GFAP+ filaments, surrounding BrdU+ cells were seen to curve and follow the route to the olfactory bulb (Fig. 8b).

In the dentate gyrus, most BrdU+ cells were nestin+ and negative for GFAP, a number located in the granule cell layer and others in the germinative sub-granular zone (Figs. 8c, d).

## Discussion

This study shows that the number of proliferating cells in the three main germinative zones: SVZ, RMS and hippocampal SGZ vary during the postnatal development from P9 and until adulthood, reaching maximal proliferating cell numbers at P12. The majority of these germinative zone BrdU+ cells showed nestin labeling and some also coexpressed GFAP. Following excitotoxic damage to the immature brain there is a significant decrease in the number of proliferating cells in the ipsilateral SVZ at all time points studied and a notable decrease within the first 3 days post lesion in the RMS, whereas no post-lesion changes are seen in the dentate gyrus. Total cell numbers were counted in the germinative zones of the brain to obtain a representative sample of cells proliferating in these areas. Noteworthy, is the significant decrease in cell number despite observed tissue shrinkage and ventricular swelling, which affect the areas analyzed.

### Changes in progenitor cell number

Proliferation studies have generally reported an increase of BrdU+ cells in the germinative zones of the brain, following injury to the adult rat brain [8,16,17]. However, the specific conclusions of the studies suggest that the response of neural progenitors to damage is variable and transient and its characteristics depend greatly on the specific germinative region, the time post-lesion and the time when BrdU is administered. Accordingly, proliferation dynamics of the germinative zones in the early postnatal brain are unique, especially during the plasticity window period, when the higher capacity for cortical reorganization and recovery could be related to a distinct behavior of progenitor pools.

Decreased SVZ proliferation in the damaged postnatal brain could be due to an increased capacity of progenitors for migration, possibly at a faster rate when compared to adults, thus leaving the SVZ "empty" or showing an apparent decrease of cells. In this sense, high-levels of polysialylated neural cell adhesion molecule (PSA-NCAM) have been shown to be present in the developing brain in progenitors of the prenatal cortex and in the developing SVZ [33]. And, high levels of PSA-NCAM are expressed in the adult cortex, subcortex and the SVZ after ischemia, suggesting a re-expression of a developmental phenotype in the injured adult brain [34]. Thus, it is likely that the postnatal brain has the capacity to upregulate migration in the germinative zones, and more so than the adult, since it has a greater capacity for regeneration and plasticity. Interestingly, TdT mediated d-UTP-biotin Nick End Labelling (TUNEL) staining shows no signs of apoptotic cell death in the SVZ (L. Acarin, unpublished results), which may suggest that BrdU immunoreactive cells of the SVZ are not dying following excitotoxic insult. This contrasts a recent study of neonatal hypoxia/ischemia in the perinatal brain where it was shown that injury resulted in depletion of the SVZ by death of neural stem cells and oligodendrocyte progenitors [28]. Migration of SVZ progenitors could also explain the results of this study showing that at 5 days post lesion and 7 days post lesion, the BrdU+ cells were found a short distance (a few nuclear diameters) away from the middle of the lateral ventricle, putatively indicating movement of progenitor cells away from the SVZ towards the striatum. In this regard, migration of progenitors towards damaged CNS parenchyma has been previously seen after chemoconvulsant-induced status epilepticus, where forebrain SVZ neurogenesis increases and a number of the neuroblasts destined for the olfactory bulb leave the RMS prematurely and migrate to the injured forebrain [13]. Likewise, it has been shown that neuroblasts having undergone division before injury can be recruited to the damaged striatum and new neurons can even be found in unaffected striatal areas [14,17]. In this study, in agreement with the hypothesis of premature deviation of progenitors from the RMS to repopulate other brain areas, a decrease of BrdU immunoreactive cells was noted in the RMS at 10 hours post lesion and 3 days post lesion, also in the absence of TUNEL staining in this area. This decrease could be due to a curtailed normal radial migration of a large quantity of cells from the SVZ to the RMS by chemoattractant molecules, which induce SVZ progenitor redirection to other brain areas that are excitotoxically damaged. Moreover, it should also be mentioned that the decrease in SVZ BrdU+ cells is no longer observed at 40 days post lesion, when the number of progenitors in the ipsilateral SVZ was not significantly different from contralateral control hemispheres. This finding further strengthens the migration or redirection theory versus cell depletion.

However, another possible explanation for the decrease in cell number seen in the SVZ, the timeframe in which gliogenesis takes place includes the first weeks of the postnatal period [35]. Glial cells generated in the SVZ migrate via radial fibers towards the cortex, striatum and dorsal white matter [36]. It is possible that NMDA-induced excitotoxic cortical damage disrupts these radial fiber directed migrational routes, preventing glial cell migration to their destined cortical zones, and inducing migration routes towards the less severely affected striatum. This could explain the disruption in nestin+ and GFAP+ prolongations leaving the SVZ and the presence of clusters of BrdU+ cells in the striatum and thalamus from 5–7 days post lesion.

The fact that no changes in the number of BrdU+ cells are observed in the SGZ of the dentate gyrus needs further elaboration. In contrast with the results of this study, insults to the adult CNS result in increases in DG neurogenesis. In cerebral ischemia, these increases are transitory, whereas a large increase in proliferating cell number is observed when BrdU is injected at 4–6 days after MCAO, while no changes are seen after 11–13 days [14,20,21,37,38]. In addition to the differences between postnatal and adult DG progenitor response to damage, it is plausible that precursors of the SVZ-RMS which are headed throughout postnatal life to give rise, for example, to late born neurons and astrocytes, and later to oligodendrocytes, would be promptly activated when cortical and striatal tissue damage is induced (as in the case of our study), whereas precursors of the SGZ would only be mobilized if massive granule cell death occurs in the DG.

### Characterization of progenitor BrdU+ cells

In general, most BrdU+ cells located in the germinative zones express the intermediate filament nestin. This protein is a typical component of the cytoskeleton of immature cells and has previously been described as a marker of non-differentiated precursor cells in the three main germinative zones [39,40]. Accordingly, the BrdU+/nestin+ population of cells seen at all time points in the SVZ may correspond to the previously described resident stem cell population that turn into rapidly proliferating precursors (most of the nestin+ cells) which express PSA-NCAM and migrate along the RMS into the olfactory cortex where they proceed to renew populations of granular and periglomular neurons and astrocytes [41,42]. In a number of recent studies, upregulation of nestin has been described after injury by ischemic insult [39,43], and traumatic brain injury [44]. It has been suggested that nestin re-expression after injury recapitulates the scenario found in the developing brain, where nestin expression commits progenitor cells to differentiate into neurons and glia, and thus plays a role in brain remodeling and recovery [39,43,44]. In addition, a population of pre-oligodendrocytes, multipolar mitotically-active late oligodendrocyte progenitors have been described to originate in the SVZ of the lateral ventricles [45] and could account for a minority of BrdU+ nuclei in the SVZ that are not labeled by nestin, GFAP or TL.

Finally, it should be noted that although the main finding of this study is the decreased number of precursor cells in the damaged SVZ, changes in the nestin+/GFAP+ prolongations were also noted. First of all, the identity of these structures is not clear. It is known that the ventricular zone (VZ) in the early postnatal brain is largely composed of radial glial cell bodies [46] and that most dividing progenitor cells in the VZ express radial glial proteins and maintain a vimentin-positive radial fiber throughout each stage of cell division [47], which plays a role in determining progenitor migration towards the cortex. Therefore, in relation to these studies, the nestin positive prolongations seen in this investigation, to be leaving the SVZ, and either crossing the corpus callosum into the cortex, along the axis of the corpus callosum, or into the striatum, are suggestive of radial glial prolongations. Furthermore, the same types of prolongations were GFAP+ and some contained GFAP+ astrocytic cells in close association with typical star shaped morphologies, further suggesting that they could be radial glia like structures directing astrocytic migration. In this sense, it has been proposed that radial glia scaffolding that directs neuronal precursor migration during embryonic development, is responsible for generating and directing late neuronal precursor migration in the neocortex [48]. Recent studies have provided in vitro and in vivo evidence that radial glia are able to generate cortical neurons [46,48-53] and that these newly generated cells take on a bipolar shape and migrate along the radial fiber of the mother cell, which remains attached to the ventricular surface [48]. These prolongations, described as radially arranged vimentin-positive processes, have also been seen to interact with cells of unipolar and bipolar morphology just above the SVZ in the corpus callosum. In more dorsal white matter, these cells showed more complicated morphologies and could be early differentiating glial cells [54]. Accordingly, some studies have also suggested that subpopulations of radial glial cells, apart from their role in neuronal guidance may be GFAP+ neural progenitor cells [55], giving rise to astrocytes, ependymal cells and also GFAP+ precursor cells. Therefore, if indeed radial glial has precursor potential, the nestin+ prolongations noted in could be radial glial progenitor cells that differentiate into the GFAP+ associated astrocytes upon leaving the SVZ.

Alternatively, because nestin is known to be re-expressed in deafferented target areas [56] and by reactive astrocytes, this finding could also suggest that progenitors are undergoing lesion-induced gliogenesis, differentiating into astrocytes that become reactive as they reach the lesioned area, as has been previously described in a study of the spinal cord, where nestin+ periventricular cells migrate to the injury site and become GFAP+ [57].

## Conclusion

The number of proliferating cells in the three main germinative zones: SVZ, RMS, and hippocampal SGZ, vary during postnatal development from P9 until adulthood, reaching maximal cell numbers at P12 and decreasing thereafter. Postnatal excitotoxic damage induces a significant decrease in the number of proliferating cells in the ipsilateral SVZ at all time points studied. A notable decrease is also observed within the first 3 days post lesion in the RMS, whereas no post-lesion changes are seen in the dentate gyrus. The majority of germinative zone BrdU+ cells are labeled for nestin and some of them coexpress GFAP. Understanding the proliferation dynamics of proliferating cells in the germinative zones of the brain in normal and pathological states is a key first step in harnessing any future potential for recovery that this endogenous population may provide.

## Methods

Long Evans black hooded rats of both sexes were used in this study. All procedures were conducted in compliance with Spanish legislation and according to the European Union directives on this subject. The animal care committee of the Autonomous University of Barcelona approved protocols.

### NMDA injections

Postnatal day 9 (P9) pups were anesthetized with isofluorane and then placed in a stereotaxic frame adapted for newborns (Kopf). The skull was opened using a surgical blade and 27 ηmoles of N-methyl-D-aspartate (NMDA; Sigma M-3262) diluted in 0.15 μl of saline solution (0.9% NaCl) were injected into the right sensorimotor cortex at the level of the coronal suture (2 mm lateral from bregma and 0.5 mm deep) with a 0.5 μl Hamilton microsyringe (needle gauge 25). After suture, the pups were placed in an incubator and maintained at 36° for 2 hours before being returned to their mothers, in order to maintain normothermia since NMDA-induced lesions are highly dependent on body temperature. In control animals the same procedure was followed but instead of a NMDA injection, pups received an injection of 0.15 μl of saline solution.

### 5'Bromodeoxyuridine (BrdU) injections

In order to examine the pattern and time course of proliferating cells in the brain BrdU, the thymidine analog which incorporates into the DNA of dividing cells during S-phase, was used to label actively proliferating cells 10 hours before sacrifice (Sigma Chemical, St Louis, MO, USA). NMDA injected, saline injected and intact controls were administered with BrdU. Pulse labelling was carried out by intraperitoneally injecting BrdU (50 mg/kg) diluted in TB (0.05 M Trizma base, pH 7.4) every 2 hours for 10 hours before sacrifice. Rats were killed at 10 hours, 3 days, 5 days, 7 days, 14 days and 40 days post-lesion. Five NMDA injected animals and two saline controls were used for each survival time. In addition, two non-lesioned rats for each survival time (aged P9, P12, P14, P16, P23 and P49 corresponding to 10 hours, 3, 5, 7, 14 and 40 days post lesion, respectively) received the same injection of BrdU and were used as control groups, in order to study changes in proliferation during development.

### Tissue processing

Rats were anesthetized and then sacrificed by intracardial perfusion for 10 minutes with 4% paraformaldehyde in 0.1 M phosphate buffer (pH 7.4). Brains were immediately removed and immersed in the same fixative for 2 hours and then cryoprotected in a 30% sucrose solution in 0.1 M phosphate buffer. Brains were then frozen with dry CO_2 _and 30 μm thick sections were cut using a cryostat (Leitz).

### Immunohistochemistry

Free-floating cryostat sections were processed for the visualization of BrdU, for the specific demonstration of proliferating cells. After endogenous peroxidase blocking with 2% H_2_0_2 _in 70% methanol for 10 minutes, DNA was denatured by first incubating in 0.082N HCl for 10 min. at 4°C and then for 30 min. in 0.82N HCl at 37°C. Sections were rinsed with TBS (0.05 M Trizma base containing 150 mM of NaCl, pH 7.4), borate buffer (pH 8.5), and 0.5% Triton X-100 in TBS (TBS-T) and then incubated in blocking buffer (BB; 10% fetal calf serum in TBS-T) for 1 hour and incubated overnight at 4°C and 1 hour at room temperature (RT) with a primary mouse anti-BrdU antibody (DAKO, Glostrup, Denmark) diluted to 1:80 in BB. After washing with TBS-T, the sections were incubated for 1 hour at RT with a secondary anti-mouse IgG biotinylated antibody (Amersham, Buckinghamshire, England, UK) in a 1:200 dilution in BB, rinsed again and incubated for 1 hour at RT in a 1:400 dilution of avidin-peroxidase (DAKO, Glostrup, Denmark) in BB. After rinsing again in TBS-T and TBS, the reaction product was detected using 50 mg of 3'3-diaminobenzidine-tetrahydrochloride (DAB, Sigma, St. Louis, MO, USA) and 33 μl of H_2_O_2 _in 100 ml of Tris buffer.

Double staining for BrdU and NeuN, GFAP and nestin was performed. Sections were first incubated with the anti-BrdU antibody overnight at 4°C and 1 hour at RT before incubation with a secondary goat anti-mouse Cy3 antibody (Amersham, Buckinghamshire, England, UK) for 1 hour at RT. Sections were then incubated overnight with either a mouse monoclonal antibody against NeuN (Chemicon, Temecula, CA, USA), a rabbit polyclonal antibody against GFAP (DAKO, Glostrup, Denmark), or a mouse monoclonal antibody against nestin (Chemicon, Temecula, CA, USA) diluted in BB at 1:500, 1:1800, and 1:1000 respectively. After rinsing, sections were incubated for 1 hour at RT with either a secondary goat anti-mouse Cy2 antibody (Amersham, Buckinghamshire, England, UK) or a secondary goat anti-rabbit Cy2 antibody (Amersham, Buckinghamshire, England, UK) diluted in BB to 1:1000. For TL histochemistry, sections processed for BrdU were incubated with a biotinylated lectin obtained from *Lycopersicon esculentum *(tomato; Sigma, St. Louis, MO) diluted to 6 μg/ml in TBS-T and then incubated for 1 hour at RT with a Cy2-conjugated streptavidin (Amersham, Buckinghamshire, England, UK) at a dilution of 1:1000.

### Image analysis & quantification

Measurements of BrdU immunoreactive cells were performed on cryostat sections (thickness of 30 μm). Sections were digitized under a 20X objective using a digital camera (Nikon Dxm1200, Tokyo, Japan) mounted on a Leitz microscope and interfaced to a PC computer using ACT1 Imaging software. Every 8th coronal section was selected from each rat for a total of 4 sections. All BrdU immunopositive nuclei were counted, using AnalySIS software (version 3.2, Soft Image Analysis System, Münster, Germany), in digitalized sections (for the best visualization and to avoid over-sampling) of the: RMS, SVZ and the entire dentate gyrus, encompassing the 2 cell body wide SGZ along the border of the granule cell layer (GCL) and the hilus. BrdU nuclei in these areas are presented as the total number of cells/section. The total number of cells in each section was averaged to obtain a mean value for each animal.

Data were analyzed using an ANOVA one-way analysis of variance followed by a Fishers test comparison. All values are presented as Mean ± Standard Error (S.E.) Statistical significance was set at P < 0.05. Because no statistically significant difference in the amount of BrdU positive cells was observed between the saline-injected and intact control animals, data from these two groups was combined to form a collective control group, which was compared to NMDA-lesioned animals.

Double stained sections were first analyzed with an optical fluorescence microscope using 10X, 20X, and 40X dry objective lenses and a 100X oil immersion objective lens. Selected sections were then analyzed and photographed using a Leica confocal microscope (TCS SP2 AOBS) where data was acquired using a 63X oil immersion lens. For sections stained with BrdU and NeuN, GFAP, TL or nestin, red (Cy3 for BrdU) and green (Cy2 for NeuN, GFAP, TL, and nestin) fluorochromes were excited by a laser beam and emissions were sequentially acquired with 2 separate photomultiplier tubes with their respective emission filters.

## Authors' contributions

MF performed the immunohistochemical study, part of the excitotoxic lesions, the quantification analysis and drafted the manuscript. LA did part of the excitotoxic lesions, helped in the analysis and interpretation of the data and the critical review of the manuscript. BC and BG coordinated the development of the study, revised the last version of the manuscript and are responsible for economic support. All authors have given final approval of the version to be published.
